# Transforming Growth Factor Beta Family: Insight into the Role of Growth Factors in Regulation of Fracture Healing Biology and Potential Clinical Applications

**DOI:** 10.1155/2015/137823

**Published:** 2015-01-29

**Authors:** Łukasz A. Poniatowski, Piotr Wojdasiewicz, Robert Gasik, Dariusz Szukiewicz

**Affiliations:** ^1^Department of General and Experimental Pathology with Centre for Preclinical Research and Technology (CePT), Second Faculty of Medicine, Medical University of Warsaw, Pawińskiego 3c, 02-106 Warsaw, Poland; ^2^Department of Rheumaorthopaedics, Institute of Rheumatology, Spartańska 1, 02-637 Warsaw, Poland; ^3^Department of Neuroorthopaedics and Neurology, Institute of Rheumatology, Spartańska 1, 02-637 Warsaw, Poland

## Abstract

The transforming growth factor beta (TGF-*β*) family forms a group of three isoforms, TGF-*β*1, TGF-*β*2, and TGF-*β*3, with their structure formed by interrelated dimeric polypeptide chains. Pleiotropic and redundant functions of the TGF-*β* family concern control of numerous aspects and effects of cell functions, including proliferation, differentiation, and migration, in all tissues of the human body. Amongst many cytokines and growth factors, the TGF-*β* family is considered a group playing one of numerous key roles in control of physiological phenomena concerning maintenance of metabolic homeostasis in the bone tissue. By breaking the continuity of bone tissue, a spread-over-time and complex bone healing process is initiated, considered a recapitulation of embryonic intracartilaginous ossification. This process is a cascade of local and systemic phenomena spread over time, involving whole cell lineages and various cytokines and growth factors. Numerous in vivo and in vitro studies in various models analysing cytokines and growth factors' involvement have shown that TGF-*β* has a leading role in the fracture healing process. This paper sums up current knowledge on the basis of available literature concerning the role of the TGF-*β* family in the fracture healing process.

## 1. Introduction

**Figure 1:**
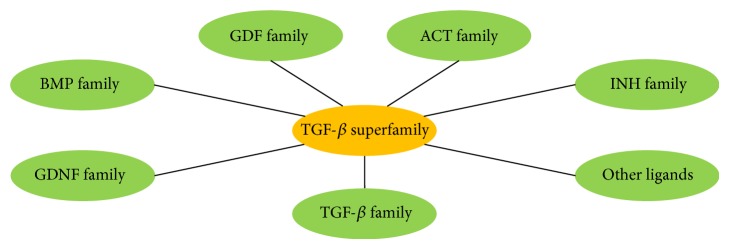
A schematic representation of TGF-*β* superfamily. TGF-*β*: transforming growth factor beta; GDF: growth and differentiation factor; ACT: activin; INH: inhibin; other ligands include Müllerian inhibiting substance (MIS) or anti-Müllerian hormone (AMH), left-right determination factor (Lefty), and nodal growth differentiation factor (Nodal); GDNF: glial-derived neurotrophic factors; BMPs: bone morphogenetic proteins.

Disorders involving the musculoskeletal system are one of the most diversified groups of diseases [1]. They include congenital and acquired diseases directly affecting bones, joints, ligaments, and muscles, as well as disorders, in which this system is involved secondarily [2]. All musculoskeletal system disorders represent a continuous challenge to the society, considering their complex and often multifactor aetiology, varied course, and economic aspects, as well as a still present problem of implementing optimal surgical and nonsurgical treatment [1, 2]. One of the most serious conditions encountered in the clinical practice is fractures, that is, breaking of the bone continuity caused by an injury or other reasons, including osteoporosis, cancer, or other systemic diseases [2, 3]. The bone damage can also be accompanied by soft tissues damage of different extent, also affecting crucial structures such as vessels and nerves [4]. Any tissue damage, caused by the injury or the surgery itself, involves not only a local immunological response and inflammation, but also a systemic immunological response related to inflow, migration, and proliferation of a broad spectrum of cells [5–9]. Cytokines are molecules responsible for controlling intracellular communication and directing the immunological reaction [10]. This group of low-molecular glycoproteins forms a “cytokine network” in the body [11, 12]. Amongst cytokines identified and described so far, a group of growth factors (GF) can also be distinguished, whose effects in certain situations can also be viewed in a context of a “growth factor network” [13]. The transforming growth factor beta (TGF-*β*) superfamily requires a particular attention. The TGF-*β* superfamily is a large and continuously expanded group of regulatory polypeptides, including a model transforming growth factor beta family and other families, such as bone morphogenetic proteins (BMPs), growth and differentiation factors (GDFs), activins (ACTs), inhibins (INHs), and glial-derived neurotrophic factors (GDNFs), as well as some proteins not included in the above families, such as Müllerian inhibiting substance (MIS), also known as anti-Müllerian hormone (AMH), left-right determination factor (Lefty), and nodal growth differentiation factor (Nodal) [14] (Figure 1).

A number of molecules in the TGF-*β* superfamily have crucial roles in tissue development and differentiation in vertebrates, control of the immunological response, and healing of tissues [14–17]. Similarly to all growth factors, the model TGF-*β* family is characterised by its pleiotropic and redundant effects, controlling its effects in most body tissues in autocrinic, paracrinic, and endocrinic ways [18, 19]. Polypeptides in the TGF-*β* family have an important role in control of cell activity and metabolism in bone and cartilage tissues throughout the ontogenetic human development [20, 21]. These attributes of the TGF-*β* family are also observed during the bone healing process, considered to be a recapitulation of embryonic intracartilaginous ossification [22, 23]. Amongst many cytokines and growth factors, the TGF-*β* family is considered to be a group playing one of numerous key functions in control of physiological phenomena during the bone healing process [24, 25]. An increased expression of ligands from the TGF-*β* family is observed both within haematoma and in serum of patients with long bone fractures [26, 27]. A broad action profile of polypeptides from the TGF-*β* family includes their effect on proliferation and differentiation of mesenchymal stem cells (MSCs), production of extracellular substance in bone and cartilage tissues, and a chemoattracting effect on a broad spectrum of cells involved in the bone healing process and the associated inflammatory response [28, 29]. In this review, we will discuss a structure of compounds in the TGF-*β* family and their relevant receptor complexes, ligand-receptor interactions, and resultant intracellular signal transmission cascades, as well as types of cellular effects in terms of their role in mechanisms and phenomena occurring during individual bone healing stages.

## 2. Structural Organization of the TGF-*β* Family

### 2.1. TGF-*β* Family Overview

For the first time, polypeptides in the TGF-*β* family were isolated by de Larco and Todaro at the end of the 1970s as a group of compounds called by them the sarcoma growth factor (SGF): the compounds able to cause malignant transformation of rat kidney fibroblasts [30, 31]. Only further studies showed that SGF is a mixture of two different compounds characterised by different properties, called transforming growth factor beta (TGF-*β*) and transforming growth factor alpha (TGF-*α*) from the epidermal growth factor (EGF) family, respectively [31, 32]. Currently, the TGF-*β* family includes its three isoforms TGF-*β*1, TGF-*β*2, and TGF-*β*3. Each of the isoforms found in humans is coded by genes having different locations in various chromosomes: in a long arm of the chromosome 19 (19q13.1) for TGF-*β*1, a long arm of the chromosome 1 (1q41) for TGF-*β*2, and a long arm of the chromosome 14 (14q24) for TGF-*β*3, respectively [33–35]. When analysing their primary structure, polypeptides from the TGF-*β* family form a highly homologous group of compounds, where mature forms of TGF-*β*1 and TGF-*β*2 are characterised by 71.4% compliance in their amino acid sequences, while TGF-*β*3 shares with TGF-*β*1 and TGF-*β*2 76% and 80% of its amino acid sequence, respectively [36, 37]. A prototype TGF-*β*1 compound, the isoform most commonly found in human tissues, in its active form after a complete posttranslation processing is a homodimer consisting of two polypeptide chains, each containing 112 amino acid residues, connected by a disulphide bond and forming a complex of a total molecular weight of 25 kDa [38, 39].

### 2.2. TGF-*β* Family Ligands Synthesis and Posttranslational Modification

**Figure 2:**
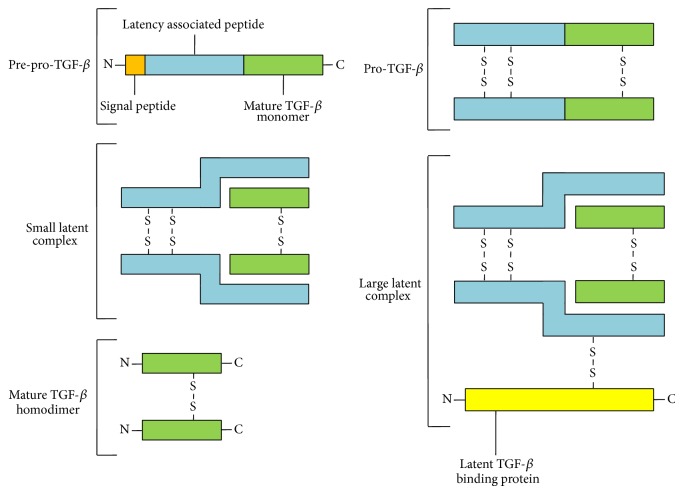
A schematic representation of TGF-*β* different forms occurring during synthesis, secretion, and activation.

Synthesis, posttranslational modification, secretion, and control of later activation of polypeptides from the TGF-*β* family form a complex and multistage process controlled by several enzymes and proteins (Figure 2).

Polypeptides from the TGF-*β* family are initially synthesised as pre-pro-TGF-*β*, a monomer of a molecular weight of ca. 55 kDa and consisting of 390 amino acid residues in total, including N-terminal signal peptide (SP) of 29 amino acids, a proregion of 249 amino acids called latency associated peptide (LAP), and a C-terminal sequence of 112 amino acids forming the actual active form of TGF-*β* after relevant modifications [40, 41]. Further stages involve proteolysis and SP removal, as well as dimerisation of two monomers with three disulphide bonds [42]. Forming of bonds is catalysed by an enzyme, disulfide isomerase (PDI), between cysteine (Cys) residues in positions 223, 225, and 356, and this way the pro-TGF*β* homodimer is created of a molecular weight of ca. 110 kDa consisting of two LAP chains and two chains of mature TGF-*β* [43, 44]. Then, created pro-TGF-*β* undergoes proteolysis by paired basic amino acid cleaving enzyme (furin, PACE) which is membrane-associated calcium-dependent serine endoprotease, abundant in the Golgi apparatus, and in consequence two connected LAP chains are separated from two connected TGF-*β* chains by cutting a bond between 278 and 279 amino acid residues [45, 46]. The proteolysis results in creation of a small latent TGF-*β* complex (SLC), in which connection between two LAP chains and two TGF-*β* chains is maintained by noncovalent bonds, despite separation of polypeptide chains [47, 48]. Furthermore, LAP chains by changes in conformation and noncovalent bonds form a specific type of protection (chaperone-like activity), maintaining TGF-*β* in its inactive form and preventing its interaction with a receptor [49]. SLC is then connected with a disulphide bond formed between cysteine residue in a 33 locations and a cysteine residue in the third of four cysteine-rich domains (8-Cys3) of the latent TGF-*β* binding protein (LTBP) of a molecular weight of 120–160 kDa, characterised, apart from its four cysteine-rich domains, by eighteen EGF-like domains; the resultant protein is called the large latent TGF-*β*1 complex (LLC) [50–53]. The next stage involves LCC secretion from a cell, and it is worth noting that LCC secretion is significantly faster than SLC secretion, and SLC not bound to LTBP is stopped at the cis pole of the Golgi apparatus [54, 55]. After secretion, parts of the complex interact with extracellular matrix (ECM) components, where C-terminal end of the LGBP protein interacts with N-terminal end of fibrylin-1, while its N-terminal end can interact with other ECM proteins, including fibronectin (FN), and this can result in its anchoring with forming of a covalent bond, with participation of a transglutaminase enzyme (TG) [56–60]. LCC anchored this way in ECM components is a form without biological activity [61]. Apart from its interactions with fibylin-1 and fibronectin, LCC can also show affinity through integrin-binding sites (RGD) in the C-terminal end of the LAP chain to integrins, glycoproteins included in adhesive proteins of heterodimeric structure consisting of two noncovalently bound subunits, one of eighteen *α* and one of eight *β* subunits [62–64]. LCC bond with integrins also allows releasing and activating the mature TGF-*β* form by changing formation of the whole complex without a need for proteolytic digestion [65–67]. The main route for TGF-*β* release from the LCC complex is related to presence and effect of numerous molecules, mainly including proteases such as plasmin, matrix metalloproteinase 2 (MMP2, gelatinase A), matrix metalloproteinase 9 (MMP9, gelatinase B), BMP-1, and others, such as thrombospondin 1 (THBS1), retinoic acid, and fibroblast growth factor 2 (FGF2), as well as reactive oxygen species (ROS); low ECM pH can also influence TGF-*β* activity [68–74].

### 2.3. TGF-*β* Family Receptors and Signalling Pathways

#### 2.3.1. TGF-*β* Family Receptors Characteristic and Regulation of Activity

**Figure 3:**
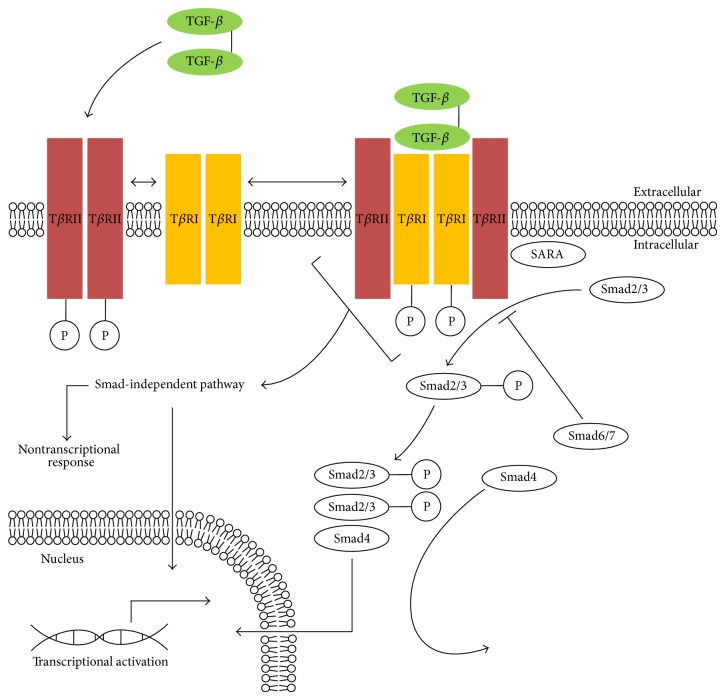
TGF-*β* associated intracellular canonical and noncanonical signaling pathways. Perpendicular line indicates an inhibitory effect; TGF-*β*: transforming growth factor beta; T*β*RI: transforming growth factor, beta receptor type I; T*β*RII: transforming growth factor, beta receptor type II; P: phosphate group; SARA: Smad anchor for receptor activation; Smad2/3: Smad family member 2/3; Smad6/7: Smad family member 6/7; Smad4: Smad family member 4.

Biological effects of homodimers, including TGF-*β*1, TGF-*β*2, and TGF-*β*3, are visible in activation of similar signalling pathways and similar cellular effects [75, 76] (Figure 3). After TGF-*β* release from ECM, it interacts with a receptor complex forming a heterotetrameric combination containing two of each of type I (T*β*RI, TGFBR1) and type II (T*β*RII, TGFBR2) subunits [76–78].

Both subunits, T*β*RI and T*β*RII, are transmembrane glycoproteins penetrating through the whole cell membrane thickness, so it is possible to distinguish their three main sections, including an N-terminal, ligand-binding extracellular part, a transmembrane part, and a C-terminal, intracellular part containing a domain with serine/threonine protein kinase activity [79, 80]. T*β*RI is a product of a gene located in a long arm of the chromosome 9 (9q22) and consists of 503 amino acid residues of a total molecular weight of 53 kDa [81–84]. The N-terminal extracellular part is located between 1 and 126 amino acid residues, the transmembrane part is located between 126 and 146 amino acid residues, and the C-terminal intracellular part is located between 146 and 503 amino acid residues [83, 84]. In the intracellular part, in the region between 175 and 205 amino acid residues, there is a so-called GS domain (TTSGSGSG), a region rich in repeated serine (Ser) and glycine (Gly) residues [83–85]. T*β*RII is a product of a gene located in a short arm of the chromosome 3 (3p22) and is larger than T*β*RI, as it consists of 567 amino acid residues of a total molecular weight of 75 kDa [84, 86–88]. The N-terminal extracellular part is located between 1 and 166 amino acid residues, the transmembrane part is located between 166 and 187 amino acid residues, and the C-terminal intracellular part is located between 187 and 567 amino acid residues [86, 89]. In its intracellular part, T*β*RII does not contain a GS domain, and its region with serine/threonine protein kinase activity shows a 41% compliance in its amino acid sequence to the domain in T*β*RI [79, 85]. A precondition for signal transduction into a cell is correct formation of a heterotetrameric receptor complex, and in particular intracellular domains with serine/threonine protein kinase activity must move closer in correct space conditions [90, 91]. First, TGF-*β* moves closer to T*β*RII subunits which, being constitutively active, undergo autophosphorylation [91–93]. The next step is phosphorylation of the GS domain forming a part of T*β*RI receptor and mutual incorporation of two T*β*RI subunits and two T*β*RII subunits, resulting in formation of a complex consisting of a TGF-*β* ligand and a receptor heterotetramer able to transmit the signal further into the cell [91]. Besides T*β*RI and T*β*RII, also a type III (T*β*RIII, TGFBR3, betaglycan) receptor can be distinguished, anchored in cell membrane with a highly glycosylated proteoglycan, of a molecular weight of 250–350 kDa [94–96]. T*β*RIII is not a typical receptor able to transmit signal because it does not contain a domain with serine/threonine activity but has a coreceptor function, able to present TGF-*β* to a complex consisting of T*β*RI and T*β*RII units and, indirectly, to modify its activity in the extracellular space [97, 98]. It has also been observed that endoglin (ENG, CD105), a homodimeric glycoprotein of a molecular weight of ca. 180–190 kDa found on cell membrane surface, has properties similar to T*β*RIII [99–101]. Endoglin also contains an RGD domain and shows affinity to TGF-*β*1 and TGF-*β*3 but not to TGF-*β*2 [100, 101]. On the other hand, T*β*RIII shows affinity to all three TGF-*β* forms and the highest to TGF-*β*2 [102]. Durability of the heterotetrameric subunit combination with the ligand is a precondition for the signal transmission into the cell [103]. Degradation of cell membrane receptors may occur in proteasome or by lysis in a lysosome [103]. T*β*RI receptor ubiquitination is catalysed by ubiquitin-activating enzyme (E1 enzyme), ubiquitin-conjugating enzyme (E2 enzyme), and ubiquitin ligase (E3 enzyme) such as Smurf1 and Smurf2 and additionally requires presence of adapter protein Smad family member 7 (Smad7), a member of the inhibitor Smad (I-Smad) subclass [104–106], whereas lysosomal degradation does not always require ubiquitination [107]. Receptor complexes and TGF-*β* ligand-bound receptor complexes are also subject to constitutive control related to their internalisation on the clathrin-dependent or lipid-raft-dependent endocytic pathway, and this ensures their correct physiological response, activity, and distribution on a cell surface [108–110].

#### 2.3.2. Intracellular TGF-*β* Canonical and Noncanonical Signalling Pathways

Intracellular signal transduction is conducted through cytoplasmic proteins, belonging to transcription factors from the Smad family [111, 112]. Currently, three Smad protein classes are distinguished, namely, receptor-regulated Smads (R-Smad) including Smad1, Smad2, Smad3, Smad5, and Smad8, common-mediator Smad (Co-Smad) including Smad4, and inhibitory Smads (I-Smad) including Smad6 and Smad7 [113]. In their structure, R-Smad and Co-Smad have similar domain structure consisting of highly conservative Mad homology 1 (MH1) at the N-terminal and Mad homology 2 (MH2) at the C-terminal, connected by a binding protein rich in proline (Pro) residues forming tridimensional globular structures [114, 115]. The MH1 domain is responsible for binding with a DNA strand, promoting transcription activity, and the MH2 domain is responsible for interactions with other proteins and oligomerisation of Smad proteins [114, 116]. Contrary to two other classes, I-Smad contains only one conservative domain MH2 [115]. In intracellular signal transmission via a canonical signalling pathway, the signal is propagated from the formed TGF-*β* ligand-bound receptor heterotetramer to the nucleus via proteins from the Smad family [117]. The activated receptor subunit T*β*RI initiates, crucial for signal transmission, phosphorylation of an R-Smad protein (Smad2 and Smad3) bound through the zinc double finger (FYVE domain) protein Smad anchor for receptor activation (SARA) [118]. SARA is a membrane-associated intracellular protein able to recruit the activated T*β*RI subunit and proteins from the Smad family dissociating from SARA following phosphorylation [118]. After phosphorylation of the C-terminal SSXS motif, a part of MH2 R-Smad proteins, Co-Smad is recruited and a heterotrimer is formed consisting of two phosphorylated R-Smads and Co-Smad [111]. This complex is transported to the nucleus, and its correct translocation is possible because of a lysine- (Lys-) rich nuclear localization-like (NLS-like) sequence forming a part of the MH1 R-Smad domain and Co-Smad and facilitates interaction with importin-*α* and importin-*β* [119, 120]. In the nucleus, the R-Smad/Co-Smad complex connects with other nuclear cofactor proteins, and the gene transcription process is initiated [121]. I-Smad proteins are responsible for negative signal transmission by competing with R-Smad proteins in binding with the receptor or Co-Smad and promote selection of receptors for proteolytic degradation [122–124]. Apart from intracellular signal transmission by Smad proteins, the TGF-*β* receptor complex can also transmit signal via a noncanonical pathway (Smad-independent pathway), that is, by other intracellular signal transmission pathways [125, 126]. The possible signal transmission pathways to the nucleus include mitogen-activated protein kinases (MAPK), such as extracellular-signal-regulated kinases 1/2 (ERK1/2), c-Jun N-terminal kinase (JNK), p38, and phosphatidylinositol-4,5-bisphosphate 3-kinase (PI3K), AKT/PKB pathway, as well as small GTP-binding proteins (Ras, RhoA, Rac1, CDC42, and mTOR) and protein tyrosine kinases (PTK2, Src, and Abl), and, furthermore, NF-*κ*B pathway and Wnt/*β*-catenin pathway [127–134].

## 3. Potential Role of TGF-*β* Family in Fracture Healing

### 3.1. Basic Principles of Fracture Healing and the Application of These Principles in Crosstalk between Cells and Growth Factors

Bone and cartilage tissues represent a special type of a dynamic microenvironment subject to constant, orderly, and lasting whole life reconstruction at a cellular level [135, 136]. A whole range of local and systemic factors are involved in maintaining metabolic homeostasis of the bone tissue so it can perform its functions [136]. When the continuity of the bone tissue is disrupted, a number of factors are activated, including a broad profile of cells and intensified gene transcription [137–139]. The whole cascade of events starts immediately with an injury and causes both local and systemic effects [7–9]. Physiological interactions and phenomena occurring during three main phases of bone healing, including an inflammatory phase, a reparative phase, and a remodelling phase, finally restore correct architecture and function of the bone tissue within 6–8 weeks [140]. During these phases, there is an interaction between various cells, whose behaviour is regulated by various cytokines, growth factors, and their dedicated receptor complexes, whose expression, participation, and function vary depending on the healing stage, and, furthermore, depends on a fracture type and kind, operative and nonoperative treatment methods applied, comorbidities, and patient's adherence to recommendations [27, 140–143]. Four main components can be distinguished in the fracture zone: periosteum, cortex, bone marrow, and surrounding soft tissues involved in repair processes; there are also differences in presence and number of individual cell types, cytokines, and growth factors in each of these components throughout the bone healing process [141, 143, 144]. The most important compounds belonging to proinflammatory cytokines, growth and differentiation factors, and angiogenic factors include the TGF-*β* family, FGF1, FGF2, platelet-derived growth factor (PDGF), insulin-like growth factor 1 (IGF-1), insulin-like growth factor 2 (IGF-2), BMP family, osteonectin (ON, SPARC), osteocalcin (BGLAP), osteopontin (OPN, SPP1), fibronectin, interleukin 1 (IL-1), interleukin 6 (IL-6), TNF-*α*, granulocyte-macrophage colony-stimulating factor (GM-CSF), macrophage colony-stimulating factor (M-CSF, CSF1), and vascular endothelial growth factor (VEGF) [144–148]. Numerous in vivo and in vitro studies on various models analysing cytokines and growth factors' involvement have shown that TGF-*β* has a leading role in the fracture healing process [24, 25, 149]. The analysis of the TGF-*β* family multidirectional effect is inseparably connected with the whole bone healing process and possible clinical application in modifying individual bone healing phases to achieve better treatment effects.

### 3.2. Expression and Localization of TGF-*β* Family during Fracture Healing

#### 3.2.1. Local Expression and Distribution of TGF-*β* in Fracture Site

Increased TGF-*β* expression, effect, and tissue distribution start with breaking of bone tissue continuity at the inflammatory phase onset, exhibiting increased local and systemic concentration [150]. TGF-*β* presence in the extracellular space with the forming haematoma can be determined in the periosteum region within 24 h from the fracture, and one of its main sources is thrombocytes, and specifically *α*-granules representing ca. 10% of their volume, as well as immune system cells such as monocytes, macrophages, and T-cells and cells directly present in the fracture region, including osteocytes, chondrocytes, and endothelial cells [150–152]. Several days after the fracture, the reparative phase is initiated, with its main stages being the intramembranous ossification phase and the endochondral ossification phase, overlapping in time. TGF-*β* presence is most pronounced during this phase within organising callus and cells found in it, such as MSCs, osteoblasts, osteocytes, chondroblasts, and chondrocytes [152, 153]. An analysis of Joyce et al. study results shows that presence of TGF-*β* RNA within the soft callus gradually increases from the 7th to 14th day of the fracture and then decreases from the 14th to 17th day of the fracture, while in the subperiosteal bone formation TGF-*β* RNA is most abundant in the 3rd to 5th day of the fracture and then it drops and reaches plateau from the 7th to 11th day of the fracture, to increase again in a period from the 11th to 15th day of the fracture; these results were additionally confirmed by observations of Bourque et al. [152, 153]. A study conducted by Matsumoto et al. showed that the increased TGF-*β* level within the callus is found between the 7th and 14th day of the fracture [154]. Si et al. showed that the increased TGF-*β* level occurs during the endochondral ossification phase [155]. The study conducted by Cho et al., concerning presence of RNA for each TGF-*β* isoform, showed that TGF-*β*1 RNA is intensively expressed during the whole healing process, while TGF-*β*2 RNA and TGF-*β*3 RNA levels are the highest on the 7th day of the fracture [156]. Meyer Jr. et al. recorded the highest TGF-*β* RNA levels within callus in a period from the 7th to 14th day of the fracture [157]. In another study, Wildemann et al. found that the TGF-*β*1 RNA level increases constitutively within callus from the 5th to 15th day of the fracture [158]. Analysing available literature and results of studies conducted in animal models concerning TGF-*β* presence and considering also slightly different methodology of each study, it can be said that the highest callus levels at the fraction site are found directly after the fracture and on onset and duration of the reparative phase; moreover, Andrew et al. found that the animal model sufficiently reflects phenomena occurring after fracture in humans [152–159]. Not only does increased expression of TGF-*β* ligands occur in callus, but it is accompanied by increased expression of T*β*RI and T*β*RII receptors and intracellular proteins such as Smad2 and Smad3, directly involved in signal transduction to the nucleus and whose increased presence is correlated with increased TGF-*β* levels [160, 161].

#### 3.2.2. Systemic Expression and Concentration of TGF-*β*

Apart from its local expression at the fracture site, TGF-*β* is also distributed systemically, which is reflected by its increased serum levels in circulating blood. Levels of circulating TGF-*β* are lower than those found at the fracture site, while its level is significantly increased versus serum collected from healthy control groups [26, 162]. It was observed that the TGF-*β* serum level increases gradually during the first two weeks reaching its maximum level on the 14th day of the fracture, and then, during the next 24 weeks, it decreases slowly reaching levels noted in patients from control groups [26, 162, 163]. Also a significant difference was observed between TGF-*β* serum levels in patients with correct bone healing and groups of patients with delayed or nonunion fracture healing [26, 162, 163]. One of the reasons, which was correlated with decreased TGF-*β* levels and delayed union, is smoking of cigarettes, where serum TGF-*β* levels in patients with fractures were significantly lower in the smoking group versus the nonsmoking patients [164–166]. Li et al. also presented a hypothesis that increased serum TGF-*β* levels in patients with close fractures may predispose to tuberculosis (MTB, TB) development [167].

### 3.3. Multiple Actions of TGF-*β* on Fracture Site Microenvironment

**Figure 4:**
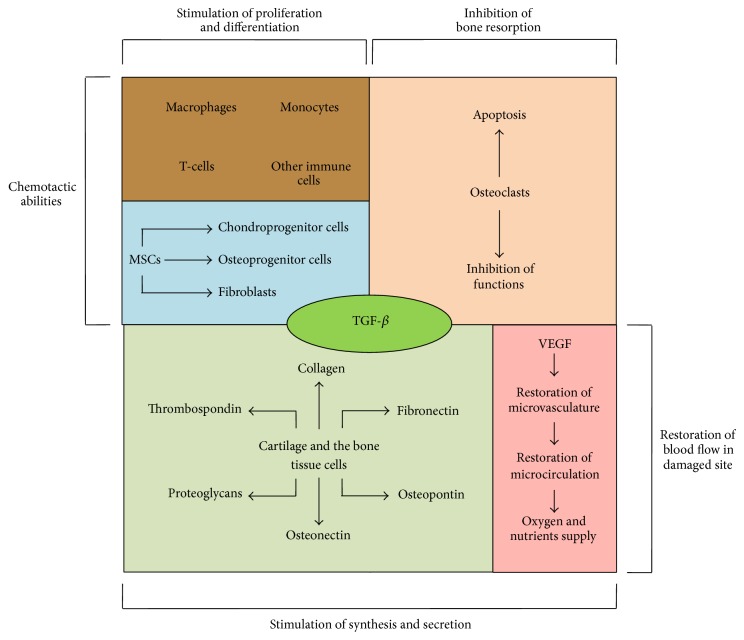
A schematic representation of TGF-*β* interactions and effects in fracture site. TGF-*β*: transforming growth factor beta; MSCs: mesenchymal stem cells; VEGF: vascular endothelial growth factor.

TGF-*β* and BMP families belong to the best known groups of compounds having the effect on the bone tissue [168]. The bone tissue is the largest TGF-*β* reservoir in the body, and it contains more than 200 *μ*g/kg of wet weight, whereas thrombocytes represent the most concentrated source of TGF-*β* around 20 mg/kg of wet weight [169, 170]. Almost every cell in a body is able to synthesise and respond to TGF-*β* ligands, and, in a case of cell lineages engaged in the bone healing process, this response also depends on differentiation degree of a relevant cell, presence of other cells, and an effect of other cytokines and growth factors. A moment when the bone tissue continuity is disrupted following an injury or osteotomy is also the moment when TGF-*β* starts to fulfill its physiological role in the processes of proliferation, differentiation, and synthesis of cartilage and bone tissue, collectively known as the bone healing process (Figure 4). Cellular effects caused by TGF-*β* attachment to cell surface can be viewed as specific connection between the inflammatory and the repair phases during fracture healing. The main sources of TGF-*β* present during the bone healing process are practically all cells involved in that process, incoming blood platelets, and the surrounding ECM releasing TGF-*β* following a mechanical injury causing tissue ischaemia and local change in pH, facilitating release not only of TGF-*β*, but also of other growth factors, such as PDGF, VEGF, or BMP-2 [171, 172]. Functionally, multidirectional TGF-*β* effects are based on autocrine and paracrine signalling and, in the cellular aspect, on induction of ECM production and ossification, resulting in bone healing. One of the most important TGF-*β* functions is its chemotactic ability, enabling recruitment of MSC, chondroprogenitor cells, osteoprogenitor cells, fibroblasts, and immune cells such as macrophages, monocytes, and T-cells [173–177]. At the same time, at early stages, TGF-*β* inhibits activation, proliferation, and differentiation of osteoclasts and moreover induces their apoptosis and additionally promotes development of callus and prevents its premature resorption, as only during the remodelling phase TGF-*β* becomes a regulator of its activity [178–181].

The TGF-*β* effect on the cartilage tissue includes proliferation of precursors or immature chondrocytes and increased ECM production in the cartilage [182]. For the bone tissue cells, TGF-*β* plays a crucial role in their proliferation and differentiation in bone development and remodelling processes. TGF-*β* controls proliferation and remodelling of osteoblasts both in vitro and in vivo, but the final result of that control also depends on the cell differentiation level and the surrounding environment. TGF-*β* effect on young forms promotes their proliferation while inhibiting terminal differentiation [183, 184]. It was also demonstrated that TGF-*β* can have a negative effect on osteocalcin and alkaline phosphatase (ALP) synthesis through osteoblasts [184, 185]. Other components also synthesised by cells of the cartilage and the bone tissues due to the TGF-*β* effect include I, II, III, V, VI, and X collagen, fibronectin, osteopontin, osteonectin, thrombospondin, proteoglycans, and alkaline phosphatase [138, 186]. Apart from secretion of components contributing to fracture healing, TGF-*β* can also influence synthesis of other growth factors such as VEGF [187, 188]. One of the most crucial components during bone healing process is redevelopment and restoration of microvasculature and microcirculation supplying oxygen and nutrients to the fracture site and creating another route for penetration by other cell types, penetrating the damaged site via blood vessels [189, 190]. TGF-*β* is also one of the angiogenic factors promoting development of new blood vessels, such as VEGF [191–194]. Full and comprehensive understanding of the TGF-*β* role in the bone healing process still poses difficulties, as often it has different and opposite cellular effects depending on numerous factors, and full interpretation of studies conducted in different models is often insufficient to consider a given effect to be typical and always occurring with the same intensity. It should also be remembered that TGF-*β* effects occur within a “growth factor network”; thus the synergistic effect is visible as a resultant cellular effect of all involved compounds.

### 3.4. Potential Clinical Approach and Evaluation of Using TGF-*β* in Fracture Healing

#### 3.4.1. Analysis of Several In Vivo Studies in Using TGF-*β* to Enhance Bone Healing

The TGF-*β* effect is continuously studied in various animal models to analyse possibilities for its use in the clinical therapies for fracture healing. Abundance and variability of methodologies in individual studies allow us to review only some of them; however, they ensure sufficient insight in the TGF-*β* effect on the fracture healing process in an animal model in various conditions, involving both small and large animals. Joyce et al. performed subperiosteal injections of TGF-*β*1 and TGF-*β*2 to newly born rats, at doses ranging from 20 to 200 ng, and they observed that subperiosteal MSC starts to proliferate and differentiate at the injection site, inducing chondrogenesis and osteogenesis, and that TGF-*β*2 has stronger effect than TGF-*β*1 [195]. Results obtained by Joyce et al. were additionally confirmed by Sun et al. in a similar experimental model [195, 196]. The experiment of Beck et al., concerning local administration of TGF-*β*1 at doses ranging from 0.5 to 5 *μ*g to rabbits with skull defect, caused stimulation, recruitment, and proliferation of osteoblasts at the defect site resulting in healing [197]. Lind et al. administered TGF-*β*1 and TGF-*β*2 with an osmotic minipump to adult rabbits with induced tibial bone fracture at a dose ranging from 1 to 10 *μ*g for six weeks, and after the end of the experiment they observed an increased mechanical strength in the fracture site and increased callus formation versus the control group not receiving TGF-*β* [198]. Nielsen et al. in their study in the rat model with local administration of TGF-*β*1 and TGF-*β*2 at the level of 4 to 40 ng/day to the fracture site in the tibial bone also observed an increased mechanical strength and callus formation at the fracture site, but only in the group receiving 40 ng/day [199]. Critchlow et al. in their study in the rabbit model with local administration of TGF-*β*2 at the level of 60 to 600 ng/day to the fracture site in the tibial bone after 14 days of observations did not note an increased mechanical strength, and callus formation at the fracture site was minimal [200]. Heckman et al. in their study in the dog model, with no healing in the radius, applied a local implant of biodegradable polymer carrier containing BMP and TGF-*β*1, depending on the group, and they found that only the carrier containing BMP had a positive effect on induction of bone growth, while the carrier containing 10 ng of TGF-*β* did not give a significant effect on the bone, also when combined with BMP at various doses [201]. Schmidmaier et al. conducted a series of experiments in animal models on combined use of TGF-*β* and IGF-1 and they demonstrated that both TGF-*β* and IGF-1 have an advantageous effect on induction of improved bone healing, but when combined that effect is significantly magnified [148, 202–204]. The analysis of a broad spectrum of experiments conducted in animal models proves that local application of TGF-*β* has a positive effect on speed and quality of resultant tissue and on completion of the bone healing process, and the final effect depends on whether TGF-*β* was supplemented systemically or locally, the form of its supplementation, and whether it was present in combination with other growth factors or specific cell populations [205–209].

#### 3.4.2. Potential Clinical Use of TGF-*β* to Enhance Human Bone Healing

The analysis of available literature does not provide a clear answer to the following question: what are the effects of therapy involving TGF-*β* on fracture healing in humans? Also effectiveness of platelet-rich plasma (PRP) administration, a blood derived preparation obtained from human blood with the increased platelet concentration, in fracture therapy, is still being studied and discussed [210–212]. PRP is a preparation containing supraphysiologic concentrations of growth factors including TGF-*β*; therefore, indirect interpretation can be attempted for the PRP effect on possible TGF-*β* as a part of previous studies on bone healing [213]. So far, a number of randomised studies in humans on PRP effectiveness in fracture therapy are scarce [210–212]. Considering the methodological limitations, interpretation of individual available results cannot ensure a sufficient statistical value allowing a clear answer on PRP effectiveness in fracture healing. Dallari et al. analysed use of PRP and lyophilized bone chips in patients undergoing high tibial osteotomy; they obtained significant increase in quantity and quality of the bone tissue versus patients administered only lyophilized bone chips [214]. A similar study on PRP use during the high tibial osteotomy procedure was conducted by Peerbooms et al., where a positive effect of combined PRP and lyophilized bone chips use was not observed [215]. Apart from the two exemplary studies described above, also other studies on PRP applications were conducted, for example, during surgical fusion of other long bones, distraction osteosynthesis, and spine fusion, which also do not provide a clear answer concerning PRP use, and to a large extent this is also limited by methodology of these studies [216–220]. Unfortunately, an analysis of an isolated TGF-*β* effect on the bone healing process in humans is currently impossible and is limited by the fact that so far no study was conducted concerning the effect of that growth factor on bone healing in humans.

#### 3.4.3. Association between Polymorphisms in the TGF-*β* Family Gene and Potential Susceptibility to Fracture

A broad spectrum of the TGF-*β* family effects on the bone tissue is also visible in a direct effect on its metabolism, involving continuous resorption and synthesis of bone structures [181]. A disturbance of a subtle balance of these processes results in osteoporosis (OP), disease related to reduced bone mineral density (BMD) [221]. Significantly increased risk of fractures and possible complications during the fracture healing process are factors inseparably related to and correlated with osteoporosis [222, 223]. Many independent authors confirm that one of the main factors correlated with that disease development is a genetic factor manifested as presence of various mutations in the human genome, including genes encoding TGF-*β* [224]. A polymorphism analysis for genes encoding TGF-*β* in various populations proves that the polymorphisms most frequently mentioned by authors and potentially related to the increased OP and, indirectly, fracture risks are T29C, C509T, T869C, G915C, and 713-8delC polymorphisms and potentially C1348T and C788T [225–230].

## 4. Conclusions and Perspective

Bone healing is a complex process involving many types of cells and their interactions mediated by cytokines and growth factors. This paper presents a current collective analysis of the possible effect of one of the most important growth factor families, TGF-*β*, on the bone healing process. It presents specifications of TGF-*β* ligands and their dedicated receptor complexes; the analysis also focused on the intracellular signal transduction pathway to the nucleus, with emphasis on possible anabolic cellular effects generated by TGF-*β* during the bone healing process. Furthermore, on the basis of current global literature, a direct and an isolated TGF-*β* effect was analysed in numerous animal models, including studies in large and small animals. The reliability of conclusions drawn on the basis of described and analysed numerous, multicenter, and independent studies by us can prove their applicability as a part of numerous methodologies. Also, a potential TGF-*β* effect on bone healing was described, as an attempt of indirect interpretation of the PRP effect as a possible TGF-*β* effect during previously conducted studies on fracture healing. Additionally, this paper notes TGF-*β* gene polymorphisms which can imply an impaired biological function of this growth factor within the bone tissue, manifested as an increased predisposition to osteoporosis. In the light of collected information, the TGF-*β* family can potentially be considered one of the most important factors stimulating and controlling the bone healing process. Although its role has been directly proven mainly in many animal models and cell cultures, it is considered that the observed TGF-*β* biological effect on bone and cartilage tissues correctly reflects its potential function in humans [159]. Currently, in the clinical practice, isolated TGF-*β* is not used in treatment of fractures or bone healing disorders. Probably, it is caused by insufficient studies on possible complications and side effects, for example, related to potential development and exacerbation of cooccurring cancer, as well as to induction of the immunosuppressive condition [231–234]. Currently, PRP is used in treatment of bone healing disorders or other diseases of the musculoskeletal system; however, results of the studies concerning effectiveness of this therapy and its long-term effects are not unambiguous. This may indicate still not fully known mechanisms of growth factors effects as a part of a “growth factor network,” meaning that it may be necessary to search for targeted fracture therapies with isolated TGF-*β*, considering results of the studies in animal models. Many independent authors emphasise a need to conduct further, more detailed studies on the TGF-*β* family participation, not only on effects or possible treatment of healing disorders, but also in terms of the widely understood regenerative medicine of other organs and tissues, to better learn and understand properties of this family [235–239]. It would enable and surely accelerate finding an answer to the question whether safe and targeted use of the therapy with isolated TGF-*β* in humans is justified. However, earlier it will not be possible to answer questions concerning full knowledge about the TGF-*β* role in the bone healing process.
